# Efficacy and safety of the switch of Triumeq® to generic (abacavir + lamivudine) + Tivicay®: data at 24 weeks

**DOI:** 10.1186/s40360-018-0252-z

**Published:** 2018-10-10

**Authors:** Julián Olalla, Javier Pérez-Stachowski, Begoña Tortajada, Alfonso Del Arco, Efrén Márquez, Javier De la Torre, Miriam Nieto, José María García de Lomas, José Luis Prada, Javier García-Alegría

**Affiliations:** 1 Internal Medicine Service, Hospital Costa del Sol, Autovía A-7, km 187, 29603 Marbella, Spain; 2 Pharmacy Service, Hospital Costa del Sol, Marbella, Spain

**Keywords:** Dolutegravir; drugs, Generic drugs, Substitution; HIV, HAART

## Abstract

**Background:**

Generic drugs may help to support antiretroviral treatment. We want to assess the efficacy and safety at 24 weeks of the change of coformulated (abacavir + lamivudine + dolutegravir) to (abacavir + lamivudine) coformulated as a generic pharmaceutical specialty + dolutegravir.

**Methods:**

Between February and June 2017, switch from Triumeq**®** to a generic pharmaceutical specialty co-formulated tablet (abacavir + lamivudine) plus Tivicay**®** was made. Demographic, viroimmunological characteristics and the Charlson index were collected. Six months after switching, efficacy and safety were evaluated.

**Results:**

Switch was made in 93 patients, with a mean age of 47 years, after six months there were five patients (5.4%) with viral loads between 50 and 400 copies, no patient had viral loads of greater amount. There were 2 interruptions due to toxicity (2.15%), in relation to symptoms of the central nervous system. There were no differences in the amount of years with HAART, nor in the previous months with the STR regimen based on abacavir + lamivudine + dolutegravir, nor in the Charlson index. The effective saving in 2017 derived from the change in these 93 patients was € 125.512.

**Conclusions:**

The change from a regimen of abacavir + lamivudine + dolutegravir seems to be safe and effective at 24 weeks.

## Background

Highly active antiretroviral therapy (HAART) based on the combination of three drugs remains the model of treatment for HIV infection. To maintain the long-term efficacy of HAART, adequate adherence over time is required [1–3]. Single Tablet Regimen (STR) suppose a greater comfort for the patient at the time of following his treatment [4, 5]. In addition, its use has been associated with a high rate of virological suppression from adherence levels of 80%, a higher adherence rate and even a lower proportion of hospitalizations and AIDS event [6–8]. The vast majority of these studies focus on the comparison of the STR of tenofovir + emtricitabine + efavirenz (TDF + FTC + EFV) versus its components separately or to other non-STR fixed dose combinations of different antiretroviral drugs [8–10]. There is no published series in real life regarding STR regimens whose third component is an integrase inhibitor in which the combination has been broken at a fixed dose.

The emergence of generic drugs in the Spanish market has posed a scenario in which it is possible to save costs at the expense of breaking STR regimes. In this context, the generic of abacavir + Lamivudine (ABC + 3TC) was commercialized in mid-2016. This allowed to have a regimen with ABC 600 mg + LMV 300 mg and dolutegravir (DTG) 50 mg more cost effective than ABC + 3TC + DTG in STR, which led to the possibility of modifying the treatment of patients undergoing treatment with the STR of ABC + 3TC + DTG to the combination of a generic pill of ABC + 3TC with a pill of dolutegravir. We report in this study the early experience (at 24 weeks) of the STR break of ABC + 3TC + DTG and its replacement by a two-tablet regimen based on a generic co-formulation of ABC 600 mg + 3TC 300 mg along with another DTG tablet 50 mg.

## Methods

A transversal retrospective analysis was made in order to examine the evolution 24 weeks after switching the antiretroviral treatment in Costa del Sol Hospital. This is a second level hospital in the southeast of Spain, with a reference population of about 400,000 inhabitants. A population of approximately 1000 patients infected with HIV is followed on a regular basis. In Spain, the economic cost of antiretroviral treatment against HIV is fully borne by the Public Health System, so that antiretroviral medication is only dispensed in hospital pharmacies. In February 2017, a systematic switch was made to the HAART of those patients under treatment with the ABC + 3TC + DTG STR to the combination of a generic co-formulation of ABC + 3TC together with another DTG tablet. Once the change was decided, it was agreed that, at the next medical visit, the prescription of the STR regimen would be changed to 2 tablets. In the Pharmacy Service, the Pharmacist reinforced the information that the doctor had given to the patient, explaining the change of HAART consisting of the modification of the number of tablets maintaining the same combination of active ingredients. In the next visit to the Pharmacy Service after the change of HAART it was verified that the patient was taking the medication correctly, as well as the absence of incidents related to it.

Since then, the prescriptions of ABC 600 mg + 3TC 300 mg + DTG 50 mg have been administered in a two-tablet regimen. Clinical follow-up did not change after the modification of the regimen, but there was a specific follow-up by the Pharmacy area.

The cost of the combination of ABC + 3TC + DTG after the break of the STR tablet is 25% cheaper than the STR. For each patient, the number of days from the change in treatment to December 31, 2017 has been calculated and multiplied by the cost difference between the two alternatives. The sum of this cost difference is the total saving since the measure began to be implemented until December 31, 2017.

The demographic and viroimmunological variables were extracted from the electronic records of the hospital, while the start data of the different antiretroviral treatments were extracted from the pharmacy’s computer system. The records were only made available to the authors. Quantitative data are shown as mean and 95% CI, and qualitative variables as percentages. SPSS 20.0 was used to analyse data.

## Results

Between February and May 2017, we proceeded to change from the STR ABC + 3TC + DTG to the generic co-formulation of ABC + 3TC together with another DTG tablet in 93 patients. The mean age was 47 years (95% CI: 45–49), with 76% of males. The level of studies was of secondary in 75%, university students in 10% and the rest with elementary studies. The mean of the Charlson index was 1.76 (95% CI: 1.23–2.30) and that of the infection age of 12.33 years (95% CI: 10.53–14.12). 48% were men who had sex with men, 34% heterosexuals and 16% intravenous drug users. 29% had suffered an AIDS event throughout the course of their disease and a quarter had suffered an AIDS event.

The mean time using HAART was 9.6 years (95% CI: 8.14–11.11), and the median number of previous ART was 1, with a median of viral failures of 0 (range: 0–2) and a median of changes due to toxicity of 1. Near 40% of patients had active smoking, 7.5% had active alcohol consumption and 5% had active drug use. 55% did not take other mediations besides ART, while 16% took statins (the most common medication after ARVs), 11% ACE inhibitors and 10% antidepressants. The most frequent comorbidities were depression and dyslipidemia (both with 11.8%), followed by High Blood Pressure (10.8%).

At the time of the change, two patients (2.2%) had viral loads between 50 and 400 copies/mL, while at 24 weeks of the change, 5 (5.4%) had viral loads between 50 and 400 copies/mL, one of these patients was detectable at the time of treatment change. Specifically, they presented values of 65, 92, 103, 118 and 259 copies/mL. Treatment was not changed in any case, remanining all of them undetectable in the next control (week 28). In the case of these patients, there were no differences in the CD4 at the time of the change, nor in the previous years with ART or the time with the STR of ABC + 3TC + DTG. The average of the Charlson index was 1.68 in the undetectables at week 24 and 3.2 in the detectable, without the difference being significant.

Before the change, the average of months with the STR of ABC + 3TC + DTG had been 12.46 (IC95%: 11.11–13.82). The adherence measured in Pharmacy before and after the change was 98%. At week 24, generic drugs were suspended in two cases (2.15%), due to toxicity (at weeks 13 and 18). The toxicity was due to symptoms of the central nervous system: a worsening of migraine and a burning mouth syndrome. In these two cases of toxicity, treatment was changed to (ABC + 3TC) + raltegravir (RTG).

Patients who discontinued due to toxicity had a higher Charlson index than the rest, although it did not reach statistical significance (2.34 vs. 1.74). No differences were observed in baseline CD4 or in the previous months with the STR of ABC + 3TC + DTG (834 vs 842 cells/microL, p n.s.). Although it did not reach statistical significance, the mean time with ART was higher in those who stopped due to toxicity (16 vs 9 years).

During 2017, there was a saving of € 125,512 with the change from one tablet per day to two tablets per day in a single shot. The estimated savings for the year 2018 of all these patients (*n* = 93), has been estimated at € 154,652 (€ 1,662 / year / patient), taking into account only the medication expenses.

## Discussion

In our series, interruptions at 24 weeks of HAART with (ABC + 3TC) + DTG were 2.15%, only attributable to toxicity. There are multiple studies that have compared antiretroviral treatments in STR versus regimens of more than one tablet, almost all of them retrospective and, always based on combinations of TDF + FTC + EFV, with very poor representation of other third agents such as rilpivirine (RPV) or elvitegravir (EVG) [8], but there is no experience communicated with the change from STR of ABC + 3TC + DTG to its components separately, as is the case of our series. In a prospective study comparing the use of STR with TDF + FTC + EFV versus the administration of its components separately no differences in efficacy were observed at 48 weeks [11]. When we compared the percentage of interruptions of the combination of (ABC + 3TC) + DTG at 24 weeks of our study with that published in the real life cohort, we observed that the percentage is similar. A Dutch cohort reported 15.3% of interruptions in non-naive patients with a median of 78 days [12], on the other hand, a German cohort reported a 7.6% interruption in treatments based on dolutegravir after 48 weeks [13]. The main cause of interruption in both cohorts were neuropsychiatric events, so that, in the German cohort, almost 80% of the interruptions due to this cause had already occurred at the end of the sixth month of follow-up. This cohort showed a percentage of interruptions at 6 months similar to those found in our patients, similar to that described in other cohort studies that have analyzed the durability of antiretroviral treatment based on integrase inhibitors [14].

No cases of interruption of the new treatment regimen due to virological failure were described, considering possible blips the five cases of viral load> 50 and < 400 copies/mL. The adherence measured in Pharmacy was not compromised either, although it is certain that, in previous studies, regimens of more than one tablet have been associated with less adherence than those of multiple tablets, with the risk of selective adherence. This fact was not observed in our cohort. On the other hand, the two cases of toxicity were related to CNS symptoms, although patients had previously tolerated the use of the ABC + LMV + DTG co-formulated tablet. It could be that both clinicians and patients were especially vigilant in the identification and interpretation of new symptoms after the change of formulations, but it could also be the case that generic pharmaceutical specialties include different percentages of impurities, as has already been possible evidenced in other fields of Medicine [15–17]. Nowadays, the most efficient antiretroviral treatment for HIV infection in Spain is, precisely, the ABC + 3TC + DTG (Triumeq®) tablet [18]. The inclusion of generic drugs in antiretroviral treatment could improve efficiency without compromising efficacy.

## Conclusion

In our experience at 24 weeks, the change from a coformulated specialty to a regimen of two tablets with the same components does not seem to have compromised the efficacy of the treatment, providing considerable financial savings. The longer term experience (at least 48 weeks) and in greater number is necessary to confirm this type of actions.

## Abbreviations

3TC: lamivudine
ABC: Abacavir
DTG: Dolutegravir
EFV: Efavirenz
HAART: Highly active antiretroviral therapy
STR: Single Tablet Regimen
TDF: Tenofovoir
