# Correction to: efficacy and safety of the switch of Triumeq® to generic (abacavir + lamivudine) + Tivicay®: data at 24 weeks

**DOI:** 10.1186/s40360-023-00693-8

**Published:** 2023-10-06

**Authors:** Julián Olalla, Javier Pérez-Stachowski, Begoña Tortajada, Alfonso Del Arco, Efrén Márquez, Javier De la Torre, Miriam Nieto, José María García de Lomas, José Luis Prada, Javier García-Alegría

**Affiliations:** 1 Internal Medicine Service, Hospital Costa del Sol, Autovía A-7, km 187, Marbella, 29603 Spain; 2 Pharmacy Service, Hospital Costa del Sol, Marbella, Spain

**Correction to:** ***BMC Pharmacol Toxicol*** **19, 63 (2018).**

10.1186/s40360-018-0252-z

The original publication of this article did not provide clear clarification that the switch from brand to a generic drug was not done by the authors for the intention of the study. Patients were informed of the options and on switching to generic drugs the patients were able to expressed their agreements or disagreements, those who expressed their disagreement continued with their regimen.

